# Free and Bound Aroma Compounds of Turnjujube (*Hovenia acerba* Lindl.) during Low Temperature Storage

**DOI:** 10.3390/foods9040488

**Published:** 2020-04-13

**Authors:** Ai-Nong Yu, Yi-Ni Yang, Yan Yang, Miao Liang, Fu-Ping Zheng, Bao-Guo Sun

**Affiliations:** 1 Beijing Advanced Innovation Center for Food Nutrition and Human Health, Beijing Technology and Business University (BTBU), Beijing 100048, China; anyu@hbmzu.edu.cn (A.-N.Y.); zhengfp@th.btbu.edu.cn (F.-P.Z.); 2 School of Chemistry & Environmental Engineering, Hubei University for Nationalities, Enshi 445000 China; yangyini2020@gmail.com (Y.-N.Y.); 2009008@hbmzu.edu.cn (Y.Y.); 201830084@hbmzu.edu.cn (M.L.)

**Keywords:** Turnjujube, *Hoveniaacerba*, volatile, aroma, bound compounds

## Abstract

Free and bound aroma volatiles from turnjujube during low temperature storage were extracted by headspace solid-phase microextraction. They were then characterized and identified using gas chromatography–mass spectrometry. Turnjujube was harvested and stored for 7, 14, and 21 days at 7 °C, the common temperature of display refrigerators in grocery stores. The results showed that 41 free and 24 bound aroma compounds were detected for the first time in turnjujube in both freshly harvested and stored turnjujube. The free and bound aroma compounds of turnjujube were markedly influenced by the storage time. The major free aroma compounds in turnjujube included esters, alcohols, aliphatic aldehydes, and aliphatic ketones. The major bound aroma compounds included borneol, eugenol, and isoeugenol, which contributed to sweet, floral, and herbaceous aroma after their hydrolysis. Freshly harvested turnjujube mostly had a fruity and herbaceous aroma, which diminished after storage at 7 °C. In contrast, the fatty aroma enhanced gradually over storage, and the floral aroma enhanced noticeably after storage for seven days. Foul odor was not detected even after storage at 7 °C for 21 days. The formation mechanisms of some aroma compounds were proposed.

## 1. Introduction

Turnjujube (*Hovenia acerba* Lindl.) belongs to the Rhamnaceae family and is distributed in China, Japan, Korea, India, and other countries. It is used as both food and as traditional Chinese medicine because of its nutritional and nutraceutical value. Its edible part is the fleshy fruit-shaped peduncle (Figure S1), which is rich in carbohydrates and various bioactive polysaccharides [1]. Polyphenols and flavones in the peduncle possess excellent antioxidant activity [2]. Because of its unique odors, excellent nutritious profile, and outstanding bioactivity, more and more studies on turnjujube have been performed to optimize its procession into juice, confiture, wine, and other kinds of food.

It is well known that volatile compounds constitute the fruit aroma, which is a crucial quality to determine fruit and its processed products. The aroma compounds in fruits can be in free forms, as well as the non-volatile bound precursors. In the precursors, volatile compounds are linked to saccharides by a glycosylated bond [3,4]. However, most free aroma compounds are easily lost, whereas some others can be enriched through biosynthesis and internal transformations during fruit processing and storage [5,6]. Therefore, both the free and bound volatile aroma compounds need to be evaluated in order to adequately appreciate fruit.

To the best of our knowledge, studies on free and bound aroma compounds of turnjujube have not been reported on. Because turnjujube ripens and deteriorates quickly at ambient temperature, storage at low temperature is effective to delay the ripening and reduce decaying after harvest. In this study, the evolution of turnjujube aroma was monitored over time with headspace solid-phase microextraction (SPME) followed by gas chromatography–mass spectroscopy (GM–MS), which is a commonly adopted technique in the analysis of fruit aroma [7,8,9]. We also analyzed the changes of the free and bound aroma compounds in turnjujube during low temperature storage and proposed the formation mechanisms of some aroma compounds. The results elucidated the composition of the volatiles and the precursors as well as their changes during the commercial shelf life of turnjujube, which could further expedite the processing and storage of turnjujube to preserve and enhance the fruit aroma.

## 2. Materials and Methods

### 2.1. Materials and Chemicals

Commercially mature turnjujube fruits were manually collected in November 2019 from the Yangtianping Village of Sancha Town in Enshi, Hubei, China (30°17’ N, 109°36’ E, 1005 m above sea level). Professor Yong-Mei Yi at the School of Forestry and Horticulture, Hubei University for Nationalities kindly identified and validated the fruits (Figure S1), then sorted and selected fruits of uniform size and color that were not damaged, shriveled or unripe.

The *n*-alkanes (C5–C22), *Aspergillus niger* pectinase (1.0 U mg^−1^), and standard compounds used for quantification and identification were obtained from Sigma-Aldrich Chemical Co. (St. Louis, MO, USA), including 4-methyl-2-pentanol (98.0%), acetoin (98.0%), isobutanol (99.8%), (E)-2-hexenal (98.0%), 1-butanol (99.0%), ethyl propanoate (99.0%), diethyl acetal (99.0%), isopentanol (98.5%), methyl salicylate (99.0%), 2-methyl-1-butanol (99.0%), isobutyric acid (99.5%), ethyl isobutyrate (99.0%), isobutyl acetate (99.0%), 2,3-butanediol (98.0%), ethyl hexanoate (99.0%), hexanal (98.0%), ethyl butanoate (99.0%), ethyl 2-butenoate (96.0%), 1-hexanol (99.9%), 2-phenylethanol (98.0%), 2-octanone (98.0%), ethyl isovalerate (99.7%), butyl isobutyrate (97.0%), methyl benzoate (99.5%), ethyl 2,4-hexadienoate (98.0%), *n*-hexyl butanoate (98.0%), ethyl benzeneacetate (99.0%), α-terpinene (89.0%), d-limonene (97.0%), γ-terpinene (97.0%), terpinolene (85.0%), (E)-pinocarveol (96.0%), camphor (95.0%), (Z)-verbenol (95.0%), 4-terpineol (95.0%), α-terpineol (95.0%), myrtenol (95.0%), verbenone (93.0%), p-ethylguaiacol (98.0%), eugenol (99.0%), isoeugenol (99.0%) and mesitylene (98.0%). Other reagents with analytical grade were from Sinopharm Chemical Reagent Co., Ltd. (Beijing, China). Double-distilled water was used throughout the experiments.

### 2.2. Extraction of Free Volatile Compounds

A similar extraction procedure for free volatile compounds from *Rubus coreanus* fruits was followed [6]. A first group of turnjujube fruit was juiced immediately with a JYZ-E16 juicer (Joyoung Co., Ltd., Jinan, China) after collection, and subsequent groups of turnjujube fruits (second, third, and fourth) were packed in polyethylene bags, tightly sealed and stored for 7, 14, and 21 days. The storage conditions were the same as the common settings of display refrigerators in grocery stores [10] to simulate shelf-life conditions (i.e., at 7 ± 0.9 °C with a relative air humidity of 64.4% ± 5.3%). After the stored fruits were also juiced for sampling, the juice was immediately centrifuged for 20 min at 4 °C and 12,298× *g* on an Avanti J-30I centrifuge (Beckman Coulter Inc., Brea, CA, USA). The supernatant (10.0 mL) was mixed with the internal standard 4-methyl-2-pentanol (0.9155 g·L^−1^ in ethanol, 20.0 μL) [11] and sodium chloride (2.0 g). The mixture was placed in a headspace extractor (20 mL) along with a magnetic stirrer, sealed with a PTFE-silicon septum, and extracted with a concept multifunctional system headspace autosampler (PAS Technology, Magdala, Germany). This autosampler was part of an Agilent 6890 N gas chromatography (GC) system coupled with an Agilent 5975i mass spectrometer (MS) (Agilent, Santa Clara, CA, USA). All these extraction procedures were done according to our previous report with DVB/CAR/PDMS SPME fiber (50/30 μm; Supelco, Bellefonte, PA, USA) for fruit juices [5]. Triplicate extractions were performed for each juice sample and all data were reported as the mean value ± standard deviation.

### 2.3. Total Soluble Solids and pH of Turnjujube Juice

Fresh juices were extracted from turnjujube fruits that had been stored for 0, 7, 14, and 21 days, and total soluble solids in these juices were analyzed with a WYT-4 digital refractometer (Beijing Yangtech Scientific Instruments Co., Ltd., Beijing, China) at 20 °C. The results were 30.6 ± 0.3%, 31.0 ± 0.2%, 29.7 ± 0.1%, and 29.5 ± 0.1%, respectively, with a mean of 30.2%. The pH values of the juice samples were determined with a PB-21 pH meter (Sartorius AG Inc., Beijing, China) at 20 °C and the results were 5.75 ± 0.09, 5.66 ± 0.07, 5.35 ± 0.09, and 4.88 ± 0.08, respectively, with a mean of 5.41. All tests were performed in triplicate.

### 2.4. Analysis of Bound Aroma Compounds

#### 2.4.1. Isolation of Bound Compounds

Clear turnjujube fruit juice (400 mL) was loaded onto a column (400 × 40 mm) packed with Amberlite XAD-2 resins (20–60 mesh, Sigma Aldrich, St. Louis, MO, USA) to extract the bound aroma compounds. The loading flow rate was at 3 mL·min^−1^. The column was first eluted with double-distilled water (800 mL at 5 mL·min^−1^) to remove acids and sugars, and then with *n*-pentane/diethyl ether (1:1, *v*/*v*, 600 mL at 5 mL·min^−1^) to remove the volatiles (i.e., the free aroma compounds). The bound components were finally collected through eluting with methanol (600 mL) at a flow rate of 5 mL·min^−1^.

#### 2.4.2. Hydrolysis of Bound Components with Enzymes

The obtained extract of bound aroma compounds was concentrated with a rotary evaporator at ≤35 °C. The residues were dissolved in 40 mL of 0.2 mol·L^−1^ phosphate −0.1 mol·L^−1^ citrate buffer (pH 5.2) and washed with 40 mL of 1:1 (*v*/*v*) *n*-pentane/dichloromethane four times in order to remove the traces of free volatiles. *Aspergillus niger* pectinase (604.0 mg) was added and mixed with the residue. The resulting mixture was divided into four headspace vials equally, which were sealed and incubated for 48 h at 37 °C, and cooled to ambient temperature. 4-Methyl-2-pentanol (0.9155 g·L^−1^ in ethanol, 20 μL), as the internal standard, was added to each headspace vial. The mixture was analyzed with GC-MS using SPME fiber as described in Section 2.2.

### 2.5. GC-MS Analysis

The methods and instruments of GC-MS analysis used in this study were similar to our previous reports [6]. Briefly, a weak polar HP-5MS capillary column (30 m × 0.25 mm i.d × 0.25 μm) was used to separate the volatile compounds. The oven temperature was programmed as follows: hold at 40 °C for 4 min, then heat to 260 °C with a rate of 5 °C·min^−1^, and then further heat to 280 °C at 15 °C·min^−1^, and finally hold at 280 °C for 1 min. A selective ion monitoring (SIM) chromatogram was extracted from the full scan chromatogram to determine the peak area. Volatile compounds were identified according to the MS data in the Nist08 and Wiley275 libraries, the LRIs in the NIST Gas Chromatography Library [12], and also compared with available standard samples. All tests were repeated twice to ensure the quality of analysis.

### 2.6. Quantification and Calculation of Odor Activity Value (OAV)

The quantification was performed according to previously published methods [5]. Briefly, a model juice solution containing sucrose (25.0 g), glucose (25.0 g), citric acid (0.001 g), and double-distilled water (460 mL) was prepared, according to the contents of acids and sugars in the turnjujube juice [2] as well as the mean values of pH and total soluble solids. The detailed data can be found in the Table S1. The concentrations of free and bound aroma compounds were converted and reported in μg·L^−1^ juice. All tests were performed in triplicate and all data were reported as the mean value ± standard deviation. The OAV was obtained by dividing the calculated concentration of volatile compound with its odor threshold in water.

### 2.7. Statistical Analyses

The significance of differences was determined by one-way ANOVA with Duncan’s multiple range test. One-way ANOVA was carried out with the IBM SPSS Statistics 25 software (IBM, Chicago, IL, USA).

## 3. Results and Discussion

### 3.1. Free and Bound Aroma Compounds

Table 1 lists the 41 free and 24 bound aroma compounds detected in turnjujube. There were seven compounds that existed in both the free form and the bound form, including 2-phenylethanol, α-terpinene, γ-terpinene, borneol, 4-terpineol, 5-caranol, and *p*-ethylguaiacol. Statistical analysis (Table 1) showed that during the extended storage at 7 °C, contents of bound α-terpineol and 5-caranol did not change significantly (*p* > 0.05). However, contents of free ethyl hexanoate, bound myrtenol, bound verbenone, and bound eugenol changed significantly (0.01 < *p* ≤ 0.05), the content of bound 2,7-dimethylocta-2,6-dienol changed very significantly (0.001 < *p* ≤ 0.01), and the contents of all other aroma compounds changed extremely significantly (*p* ≤ 0.001).

#### 3.1.1. Alcohols, Aliphatic Aldehydes, and Ketones

All seven alcohols identified from the aroma of turnjujube existed as free volatiles, and 2-phenylethanol existed in both free and bound forms. Among the free alcohols, the content of 1-butanol, 2-methyl-1-butanol and 2,3-butanediol increased with storage time, while the content of 1-hexanol decreased. The change of other alcohols did not show a clear pattern that could be related to storage time. The contents of isobutanol, 1-butanol and 1-hexanol remained high after harvesting and storage. The freshly harvested turnjujube possessed abundant (listed in decreasing order of content) 1-hexanol, isobutanol, isopentanol, 1-butanol, and 2-phenylethanol. The C6 alcohol 1-hexanol was released from green plant tissue after mechanical damage [11] and related to herbaceous and floral aroma [13].

Additionally, 4 aliphatic aldehydes and 3 aliphatic ketones were found in the free volatiles from the aroma of turnjujube, and no aliphatic aldehyde or ketone was detected in the bound compounds. The results were in agreement with reported data of blackcurrant [8]. Among these compounds, the contents of 3-hydroxybutanal, acetoin, and 2-octanonewere increased along the storage time, while contents of 2,6,8-trimethyl-4-nonanone and (E)-2-hexenal decreased. The C6 compounds (E)-2-hexenal and hexanal, which generally gave herbaceous aroma to fruits, remained abundant at all stages, except that (E)-2-hexenal was not detected after 21 days of storage. In contrast, 2-butenal was not detected in the newly harvested fruit but its content increased to 443.38–609.27 μg·L^−1^ after seven days of storage. The content of acetoin remained high throughout all stages and was increased with storage time, which reached 2591.83 and 7746.04 μg·L^−1^ after 14 and 21 days, respectively. Similar results were reported previously in which high acetoin (2684 μg·L^−1^) from Zixiang seedless raisins was found [14].

Among these compounds found in turnjujube juice, C4–C5 alcohol, aldehydes, and ketones were possibly formed through the enzymatic conversion of fatty acids or amino acids [15]. According to the reported generation mechanism of C6 alcohols and aldehydes, including 1-hexanol, hexanal, and (E)-2-hexenal [5,6], they were presumably generated from the reactions of unsaturated linoleic and linolenic acids catalyzed by lipoxygenase and hydroperoxydelyase.

#### 3.1.2. Esters

Esters are known to give fruity and sweet aroma to fruits [11,16]. A total of 15 esters were detected in turnjujube aroma. Thirteen of them were in free volatiles. The other two were not found in the free volatiles and in the bound aroma compounds and were both aromatic esters (i.e., methyl benzoate and methyl salicylate). Esters accounted for 25.9% of all detected aroma compounds and the number of detected compounds was less than that of terpenoids. The total concentration of free esters declined over storage. Specifically, the total free esters in turnjujube stored for 0, 7, 14, and 21 days reached 9095.73, 6664.07, 3439.28, and 795.80 μg·L^−1^, respectively. This loss could be attributed to evaporation during storage because these esters were highly volatile [17].

However, certain compounds became more abundant during the extended storage time, including free isobutyl acetate and ethyl hexanoate. Additionally, free ethyl butanoate and ethyl 2,4-hexadienoate were not detected from freshly harvested turnjujube and only appeared after storage. It could be inferred that turnjujube, which is rich in carbohydrates, produced alcohols and acids during its metabolism during storage, which then generated the corresponding esters via enzymatic catalysis [18,19]. Four ethyl esters in the free volatiles, including ethyl propanoate, ethyl isobutyrate, ethyl 2-butenoate, and ethyl isovalerate, were also found with high concentrations both upon harvesting and after the storage, and they were the predominant free esters in the aroma of turnjujube. These results were similar to that in the Oregon Chickasaw blackberry odor because ethyl propanoate and ethyl 2-butenoate were also its important components [20], and that in mango because ethyl isobutyrate and ethyl isovalerate were its major aroma compounds [21].

Furthermore, only methyl benzoate and methyl salicylate were detected as bound esters, both of which declined along the extended storage. Similar observations were made in cherry, where these compounds alone form the greatest part of all bound esters [11].

#### 3.1.3. Terpenoids

Terpenoids are the most important aroma compounds that determine the typical feature of fruits [22]. In turnjujube, a total of 22 terpenoids were identified and quantified, including four monoterpene hydrocarbons and 18 oxygenated monoterpenes. Terpenoids were the predominant compounds detected from turnjujube aroma (Table 1). According to reported results [23,24], terpenoids and esters also played important roles in the odor profile of currant products.

**Figure 1:**
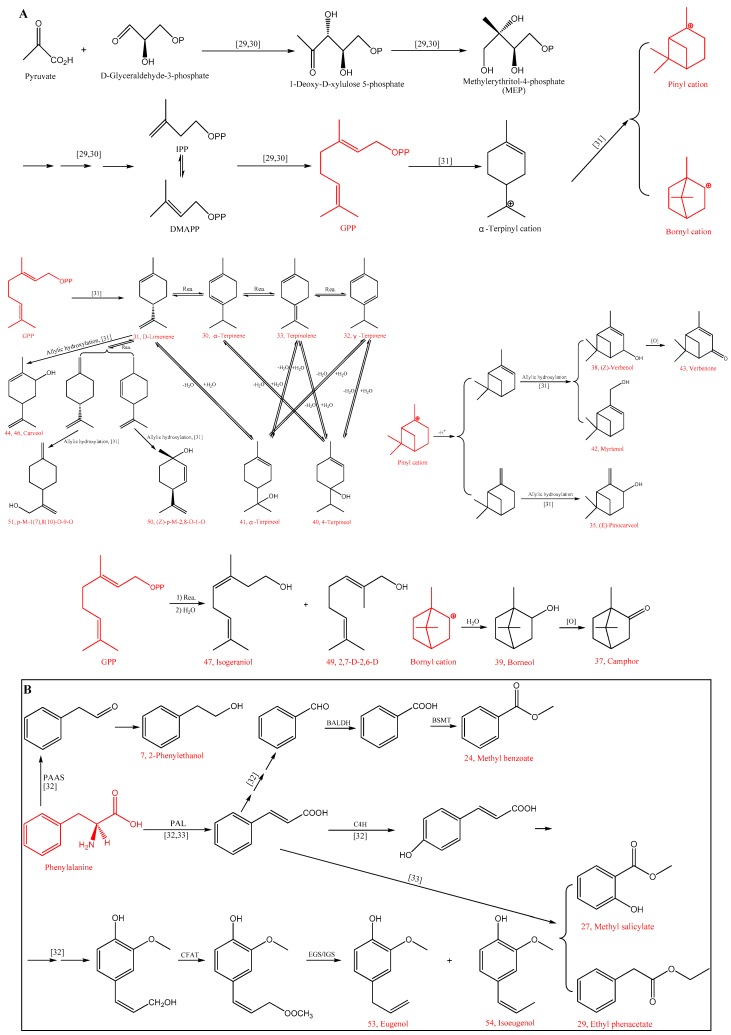
Proposed formation pathway of some aroma compounds in turnjujube: **A**—terpenoids, **B**—benzenoid aroma compounds. Symbols in the abbreviations are as follows: Rea.: rearrangement; [O]: oxidation; BALDH: benzaldehyde dehydrogenase; BSMT: benzoic acid/salicylic acid carboxyl methyltransferase; PAAS: phenylacetaldehyde synthase; PAL: L-phenylalanine ammonia lyase; C4H: cinnamate-4-hydroxylase; CFAT: coniferyl alcohol acetyltransferase; EGS: eugenol synthase; IGS: isoeugenol synthase. Compound numbers correspond to Table 1.

All of the 4 monoterpene hydrocarbons (α-terpinene, d-limonene, γ-terpinene and terpinolene) were found in the free volatiles, while only α-terpinene and γ-terpinene were found in the bound compounds. The storage time increased the contents of free and bound α-terpinene, free γ-terpinene, and free terpinolene, although their contents were always low in comparison to oxygenated monoterpenes. It could be speculated that these monoterpene hydrocarbons might have resulted from the dehydration of related monoterpenols (Figure 1A) [5].

Eighteen oxygenated monoterpenes mostly existed in the form of bound compounds, and only five oxygenated monoterpenes were found in the free volatiles, including (E)-sabinene hydrate, camphor, borneol, 4-terpineol, and 5-caranol. A total of 16 oxygenated monoterpenes were found in the bound compounds, and borneol, 4-terpineol and 5-caranol were found in both forms. These results resembled the odor profile of jujube, which also belonged to the Rhamnaceae family along with turnjujube. In the jujube aroma, d-cadinene was detected as the sole terpenoid in the free volatiles (bound terpenoids were not examined in this study) [25], and free terpenoids were not found in the beverage fermented from jujube [26]. Among the detected oxygenated monoterpenes in the free volatiles, only camphor was found in freshly harvested turnjujube, 4-terpineol was detected after 14 days of storage, and borneol was detected (9.49 μg·L^−1^) after 21 days of storage. The storage procedure increased contents of (E)-sabinene hydrate and 4-terpineol in the free volatiles.

Additionally, borneol was detected in the aroma of turnjujube at all stages and its content (1636.44–4177.34 μg·L^−1^). It always exceeded that of all other bound oxygenated monoterpenes among the 16 oxygenated monoterpenes in the bound compounds, in agreement with reports on the blackcurrant cultivar “Sofya” and the Satsuma mandarin fruit [8,9]. Bound borneol could generate herbaceous odor after hydrolysis [27] and thus effectively contribute to the turnjujube aroma. Other highly abundant oxygenated monoterpenes in the bound compounds included (E)- and (Z)-carveol, isogeraniol, 5-caranol, and 2,7-dimethylocta-2,6-dienol. Among them, the detection of both (*Z*)-carveol and isogeraniol resembled the situation of blackcurrant cultivars. Specifically, both compounds were not detected in the free volatiles, but were readily found in the bound compounds [8].

Among terpenoids, only monoterpenes (C10) were detected in this study, most likely generated via the methylerythritol-4-phosphate (MEP) pathway [28]. In Figure 1A the possible mechanism for the formation of these monoterpenes is given with reference of literatures [29,30,31].

#### 3.1.4. Others

Other compounds identified in the turnjujube aroma were mainly phenols and acetals. Among the three detected phenols, p-ethylguaiacol appeared only after 14 days of storage, and was detected in both the free volatiles and the bound compounds although at very low content (0.56–2.29 μg·L^−1^). The other two phenols (i.e., eugenol and isoeugenol), were not found in the free volatiles but were abundant in the bound compounds (356.07–2109.76 μg·L^−1^), which was similar to a report concerning *Rubus corchorifolius* [5]. Both eugenol and isoeugenol made important contributions to the turnjujube aroma after hydrolysis because they both had a low odor threshold of 6 μg·L^−1^ in water (Table 2). The two detected acetals, 2,4,5-trimethyl-1,3-dioxolane and diethyl acetal, only existed in the free volatiles. They were formed from the condensation between acetaldehyde and 2,3-butanediol or ethanol. Diethyl acetal was not found in the freshly harvested sample but became abundant (109.22–146.38 μg·L^−1^) after seven days of storage. This was reported previously in Baijiu [32] as a result of fermentative processes, which may also take place in turnjujube due to its high carbohydrate content. Isobutyric acid and mesitylene were also found in the free volatiles.

There were seven benzenoid aroma compounds in the detected aroma compounds, including one alcohol (2-phenylethanol), three esters (methyl benzoate, methyl salicylate, and ethyl benzeneacetate), and three phenols (*p*-ethylguaiacol, eugenol, and isoeugenol). Figure 1B shows the formation mechanism of these compounds, which have been suggested to be derived from phenylalanine after a series of enzymatic reactions [39,40].

### 3.2. Changes of Odor Profiles

Table 2 summarizes the OAVs for the compounds of turnjujube aroma, which were calculated from the reported odor thresholds in water. The OAVs of the free volatiles in the aroma of freshly harvested turnjujube fell in the following order: esters (total OAVs of 30766.65) > aliphatic aldehydes and ketones; (total OAVs of 340.91) > alcohols (total OAVs of 77.122); and terpenoids, phenols, and acetals made negligible contributions. The free volatiles that contributed significantly to the aroma of stored turnjujube included esters (total OAVs of 672.70–17383.71); aliphatic aldehydes and ketones (total OAVs of 2239.69–2270.02); alcohols (total OAVs of 57.27–64.27); acetals (total OAVs of 22.31–29.87); and terpenoids (total OAVs of 1.10–1.95). Only free phenols (total OAVs of 0.0063–0.026) made negligible contribution to the aroma of stored turnjujube. These results showed that the major free volatiles in the aroma of freshly harvested and stored turnjujube included esters, alcohols, aliphatic aldehydes, and ketones, all of which made important contributions to the turnjujube aroma. Freshly harvested (0 day) turnjujube mainly received its aroma from ethyl isobutyrate (OAV of 30411.73), hexanal (OAV of 315.54), and ethyl isovalerate (OAVof272.84). During cold storage, ethyl isobutyrate emission decreased steeply, although remaining the main contributor to overall aroma during 14 days of storage. Similarly, ethyl isovalerate decreased in the observation period. On the other hand, compounds like 2-butenal or acetoin were scarcely detectable or not detectable at all in freshly harvested fruit, but were important contributors to stored fruit aroma. Although isobutanol, 1-butanol and 1-hexanol were abundant at all stages, they did not contribute significantly to the aroma because their odor threshold was high (Table 2).

Although the bound compounds do not directly contribute to the aroma of turnjujube, they release volatile compounds through enzymatic or acidic hydrolysis during storage to contribute to the odor. The bound compounds of turnjujube aroma mainly included borneol (OAV of 11.69–29.84), eugenol (OAV of 264.52–351.63), and isoeugenol (OAV of 59.35–97.95). Their contents thus influenced the odor profile of turnjujube during storage and after processing due to hydrolysis. Other bound compounds had negligible contribution to the turnjujube aroma.

**Figure 2:**
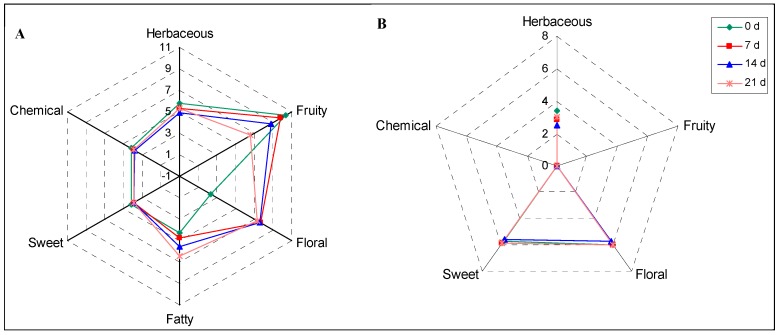
The aromatic series of (**A**)—free and (**B**)—bound aroma compounds in turnjujube according to its odor activity values, 1, 2. 3…11: logarithmic scale.

The odor profile of turnjujube could be established by grouping aroma compounds according to their odor types to thus convert the quantitative data into more straightforward sensory perception, similar to other fruits such as raisin, grape, and cherry [11,27,34]. The detected aroma compounds in turnjujube were grouped into six types, including herbaceous, fruity, floral, sweet, fatty, and chemical (the type of odor completely opposite to the odor of turnjujube which is a foul odor) (Table 2). Figure 2 shows the odor vectors of the free and bound aroma compounds, which are plotted based on their OAVs in natural logarithmic scale [11] and demonstrates the overall aroma characteristics of turnjujube and the aroma variation upon storage. Figure 2A shows that freshly harvested turnjujube is mainly fruity and herbaceous, but these odors diminish along the prolonged storage at 7 °C while the fatty aroma is gradually strengthened. Additionally, freshly harvested turnjujube was only weakly floral, but the floral odor was improved notably after seven days of storage and did not vary significantly after seven days. This was because 2-butenal, which had a characteristic floral smell [36], increased remarkably after seven days of storage and its OAV varied very little upon further storage. Figure 2A also shows that the sweet and chemical odors change very little throughout the 21 days and are only slightly stronger upon initial harvesting. These results indicated that the storage up to 21 days would not increase the undesired chemical smell of turnjujube.

As shown in Figure 2B, the predominant bound aroma compounds in turnjujube (i.e., borneol, eugenol, and isoeugenol) were herbaceous, floral, and sweet [5,11,27]. The herbaceous, floral, and sweet odors of turnjujube were strengthened after enzymatic or acidic hydrolysis during storage and processing. The odor variation was very limited after 21 days of storage, except that the herbaceous odor was slightly stronger when the fruit was freshly harvested.

## 4. Conclusions

In conclusion, 41 free and 24 bound aroma compounds were detected for the first time in both freshly harvested turnjujube and stored turnjujube. Esters, alcohols, aliphatic aldehydes, and aliphatic ketones appeared to be the major free aroma compounds, and they also made important contributions to the turnjujube aroma. Borneol, eugenol, and isoeugenol appeared to be the major bound aroma compounds, and they appeared to be abundant both upon harvest and after storage. Additionally, they also released volatile compounds that contributed to the sweet, floral, and herbaceous odor after their hydrolysis. The fruity and herbaceous odor of freshly harvested turnjujube diminished upon storage and the fatty odor was strengthened. The floral odor increased significantly after seven days of storage, and no foul odor was detected after 21 days of storage.

## Figures and Tables

**Table 1:** Free and bound aroma compounds of turnjujube after storage at 7 °C.

Nos	RI	Compounds	ID	Free-Form (μg·L^−1^)					Bound-Form (μg·L^−1^)				
				0 d	7 d	14 d	21 d	Sig	0 d	7 d	14 d	21 d	Sig
1	602	Isobutanol	A	1199.61 ± 35.68	984.73 ± 27.95	2106.62 ± 128.47	3150.69 ± 384.48	***	ND	ND	ND	ND	–
2	648	1-Butanol	A	146.29 ± 9.72	561.11 ± 15.60	698.33 ± 28.75	994.13 ± 39.58	***	ND	ND	ND	ND	–
3	728	Isopentanol	A	265.16 ± 12.55	197.84 ± 6.23	185.69 ± 5.39	198.48 ± 12.85	***	ND	ND	ND	ND	–
4	732	2-Methyl-1-butanol	A	ND	100.40 ± 2.40	102.87 ± 5.76	160.63 ± 14.53	***	ND	ND	ND	ND	–
5	793	2,3-Butanediol	A	ND	ND	144.73 ± 5.31	725.04 ± 44.60	***	ND	ND	ND	ND	–
6	872	1-Hexanol	A	5130.94 ± 140.54	1547.69 ± 41.67	1173.75 ± 42.28	804.14 ± 48.25	***	ND	ND	ND	ND	–
7	1113	2-Phenylethanol	A	40.78 ± 5.27	138.02 ± 8.53	107.68 ± 7.74	162.59 ± 14.55	***	117.18 ± 5.01	58.24 ± 2.62	73.20 ± 0.57	46.97 ± 5.42	***
		***Alcohols***		***6782.78***	***3529.79***	***4519.67***	***6195.7*** ***0***		***117.18***	***58.24***	***73.20***	***46.97***	
8	631	2-Butenal	B	ND	609.27 ± 21.00	578.82 ± 14.21	443.38 ± 10.55	***	ND	ND	ND	ND	–
9	687	3-Hydroxybutanal	C	ND	ND	295.15 ± 10.27	439.71 ± 28.87	***	ND	ND	ND	ND	–
10	802	Hexanal	A	757.30 ± 86.78	441.64 ± 32.50	303.39 ± 20.87	500.14 ± 62.79	***	ND	ND	ND	ND	–
11	854	(E)-2-Hexenal	A	294.56 ± 60.96	212.02 ± 16.08	96.23 ± 12.50	ND	***	ND	ND	ND	ND	–
		***Aliphatic aldehydes***		***1051.86***	***1262.93***	***1273.59***	***1383.23***		***0***	***0***	***0***	***0***	
12	708	Acetoin	A	112.62 ± 11.40	596.16 ± 17.52	2591.83 ± 418.73	7746.04 ± 361.19	***	ND	ND	ND	ND	–
13	872	2-Octanone	A	ND	2.55±0.13	2.55±0.083	3.85±0.24	***	ND	ND	ND	ND	–
14	1212	2,6,8-T-4-N	C	34.76 ± 2.91	28.39 ± 3.43	25.67 ± 0.54	ND	***	ND	ND	ND	ND	–
		***Aliphatic ketones***		***147.38***	***627.1***	***2620.05***	***7749.89***		***0***	***0***	***0***	***0***	
15	711	Ethyl propanoate	A	801.20 ± 33.42	867.05 ± 4.63	624.10 ± 37.16	ND	***	ND	ND	ND	ND	–
16	753	Ethyl isobutyrate	A	6690.58 ± 390.37	3705.59 ± 174.25	1364.58 ± 117.72	138.64 ± 28.82	***	ND	ND	ND	ND	–
17	771	Isobutyl acetate	A	ND	33.94 ± 1.96	42.30 ± 4.73	77.94 ± 11.16	***	ND	ND	ND	ND	–
18	805	Ethyl butanoate	A	ND	232.54 ± 9.87	183.69 ± 12.74	ND	***	ND	ND	ND	ND	–
19	848	Ethyl 2-butenoate	A	587.42 ± 27.49	1077.44 ± 27.04	803.71 ± 50.65	327.92 ± 23.11	***	ND	ND	ND	ND	–
20	857	Ethyl isovalerate	A	818.53 ± 32.66	575.23 ± 36.60	278.38 ± 17.59	112.10 ± 9.46	***	ND	ND	ND	ND	–
21	919	Butyl isobutyrate	A	18.92 ± 0.48	15.33 ± 1.17	ND	ND	***	ND	ND	ND	ND	–
22	956	E 3-H-3-M	B	43.61 ± 1.61	29.00 ± 2.68	36.88 ± 1.62	34.56 ± 1.87	***	ND	ND	ND	ND	–
23	1003	Ethyl hexanoate	A	6.60 ± 0.18	7.15 ± 0.58	7.79 ± 0.47	8.14 ± 0.29	*	ND	ND	ND	ND	–
24	1095	Methyl benzoate	A	ND	ND	ND	ND	–	0.67 ± 0.03	0.21 ± 0.00	0.22 ± 0.00	ND	***
25	1099	Ethyl 2,4-hexadienoate	A	ND	26.14 ± 1.44	18.50 ± 1.72	11.41 ± 1.37	***	ND	ND	ND	ND	–
26	1151	*n*-Hexyl butanoate	A	18.46 ± 1.30	ND	ND	ND	***	ND	ND	ND	ND	–
27	1195	Methyl salicylate	A	ND	ND	ND	ND	–	9.51 ± 1.14	2.51 ± 0.07	ND	ND	***
28	1231	Linalyl formate	B	25.21 ± 0.53	16.20 ± 0.55	25.94 ± 0.87	23.40 ± 0.82	***	ND	ND	ND	ND	–
29	1247	Ethyl benzeneacetate	A	85.20 ± 2.48	78.46 ± 2.22	53.41 ± 2.31	61.69 ± 4.29	***	ND	ND	ND	ND	–
		***Esters***		***9095.73***	***6664.07***	***3439.28***	***795.8*** ***0***		***10.18***	***2.72***	***0.22***	***0***	
30	1016	α-Terpinene	A	ND	11.66 ± 0.36	14.08 ± 0.57	16.88 ± 0.64	***	ND	ND	0.86 ± 0.02	0.87 ± 0.05	***
31	1029	d-Limonene	A	ND	7.78 ± 0.08	11.60 ± 0.93	8.98 ± 0.15	***	ND	ND	ND	ND	–
32	1060	γ-Terpinene	A	9.29 ± 0.07	11.83 ± 0.26	23.43 ± 1.06	29.23 ± 1.56	***	ND	1.02 ± 0.02	1.24 ± 0.06	1.15 ± 0.06	***
33	1088	Terpinolene	A	ND	5.61 ± 0.12	6.62 ± 0.17	7.65 ± 0.32	***	ND	ND	ND	ND	–
		***Monoterpene hydrocarbons***		***9.29***	***36.88***	***55.73***	***62.74***		***0***	***1.02***	***2.10***	***2.02***	
34	1124	6-Camphenol	B	ND	ND	ND	ND	–	ND	3.45 ± 0.31	3.64 ± 0.76	4.23 ± 0.35	***
35	1139	(E)-Pinocarveol	A	ND	ND	ND	ND	–	12.07 ± 0.16	9.26 ± 2.31	ND	ND	***
36	1140	(E)-Sabinene hydrate	B	ND	17.29 ± 0.87	21.28 ± 0.98	30.34 ± 2.54	***	ND	ND	ND	ND	–
37	1145	Camphor	A	1.80 ± 0.09	9.10 ± 0.36	15.75 ± 0.85	10.96 ± 1.07	***	ND	ND	ND	ND	–
38	1149	(Z)-Verbenol	A	ND	ND	ND	ND	–	23.64 ± 0.18	16.13 ± 0.88	13.23 ± 3.04	2.16 ± 0.21	***
39	1166	Borneol	B	ND	ND	ND	9.49 ± 2.64	***	4177.34 ± 14.88	2300.27 ± 184.72	1636.44 ± 46.46	2749.00 ± 69.10	***
40	1178	4-Terpineol	A	ND	ND	10.55 ± 2.77	69.43 ± 5.75	***	ND	2.08 ± 0.19	7.77 ± 0.24	6.70 ± 0.19	***
41	1191	α-Terpineol	A	ND	ND	ND	ND	–	4.76 ± 1.04	3.06 ± 0.41	3.75 ± 0.58	4.31 ± 0.37	ns
42	1197	Myrtenol	A	ND	ND	ND	ND	–	4.06 ± 0.72	3.22 ± 0.15	2.75 ± 0.098	2.84 ± 0.13	*
43	1210	Verbenone	A	ND	ND	ND	ND	–	15.87 ± 0.64	17.15 ± 0.21	13.45 ± 1.69	14.98 ± 1.12	*
44	1217	(E)-Carveol	A	ND	ND	ND	ND	–	38.48 ± 2.38	14.27 ± 0.99	19.59 ± 3.67	20.13 ± 0.77	***
45	1229	(Z)-2-(3,3-D) E	B	ND	ND	ND	ND	–	14.19 ± 0.94	9.78 ± 0.40	7.94 ± 0.70	12.57 ± 0.28	***
46	1232	(Z)-Carveol	B	ND	ND	ND	ND	–	70.54 ± 2.05	43.36 ± 1.66	35.83 ± 0.89	39.64 ± 1.62	***
47	1235	Isogeraniol	B	ND	ND	ND	ND	–	29.29 ± 2.03	21.66 ± 0.14	14.18 ± 0.72	20.99 ± 0.16	***
48	1244	5-Caranol	C	ND	33.91 ± 2.35	23.60 ± 1.65	1.89 ± 0.21	***	55.74 ± 7.20	48.00 ± 8.36	35.49 ± 5.59	49.02 ± 2.67	ns
49	1256	2,7-D-2,6-D	C	ND	ND	ND	ND	–	47.92 ± 4.87	40.28 ± 0.16	30.68 ± 2.52	40.63 ± 1.38	**
50	1293	(Z)-p-M-2,8-D-1-O	C	ND	ND	ND	ND	–	11.91 ± 0.04	8.73 ± 0.81	8.30 ± 0.05	8.66 ± 0.29	***
51	1311	p-M-1(7),8(10)-D-9-O	C	ND	ND	ND	ND	–	13.44 ± 1.03	9.76 ± 1.09	7.58 ± 0.77	10.65 ± 0.14	***
		***Oxygenated monoterpenes***		***1.8*** ***0***	***60.3*** ***0***	***71.18***	***122.11***		***4519.25***	***2550.46***	***1840.62***	***2986.51***	
52	1281	p-Ethylguaiacol	A	ND	ND	0.56 ± 0.00	2.29 ± 0.13	***	ND	ND	ND	2.14 ± 0.23	***
53	1360	Eugenol	A	ND	ND	ND	ND	–	1800.59 ± 95.60	2109.76 ± 244.02	1587.11 ± 119.16	2086.71 ± 106.93	*
54	1452	Isoeugenol	A	ND	ND	ND	ND	–	587.72 ± 24.79	395.71 ± 24.29	356.07 ± 18.90	382.02 ± 10.72	***
		***Phenols***		***0***	***0***	***0.56***	***2.29***		***2388.31***	***2505.47***	***1943.18***	***2470.87***	
55	723	2,4,5-T-1,3-D	B	ND	ND	12.80 ± 0.49	132.17 ± 8.13	***	ND	ND	ND	ND	–
56	726	Diethyl acetal	A	ND	129.08 ± 2.53	109.22 ± 7.15	146.38 ± 4.21	***	ND	ND	ND	ND	–
57	748	Isobutyric acid	A	ND	32.68 ± 3.29	143.68 ± 8.30	ND	***	ND	ND	ND	ND	–
58	969	Mesitylene	A	66.28 ± 5.03	39.57 ± 4.30	36.27 ± 2.42	22.4 7 ± 2.62	***	ND	ND	ND	ND	–
		***Others***		***66.28***	***201.33***	***301.97***	***301.02***		***0***	***0***	***0***	***0***	

Nos.: numbers; RI: retention index. ID stands for the reliability of the identification proposal: A, identified, mass spectrum, RI agreed with literature data [12] and RI agreed with standards; B, tentatively identified, mass spectrum agreed with the mass spectral database and RI agreed with literature data [12]; C, tentatively identified, mass spectrum agreed with the mass spectral database. Sig stands for statistical significance: *, significant at *p* ≤ 0.05; **, significant at *p* ≤ 0.01; ***, significant at *p* ≤ 0.001; ns, not significant (*p* > 0.05). ND: not detected. 2,6,8-T-4-N: 2,6,8-trimethyl-4-nonanone; E 3-H-3-M: ethyl 3-hydroxy-3-methylbutanoate; (Z)-2-(3,3-D) E: (Z)-2-(3,3-dimethylcyclohexylidene)ethanol; 2,7-D-2,6-D: 2,7-dimethylocta-2,6-dienol; (Z)-p-M-2,8-D-1-O: (Z)-p-mentha-2,8-dien-1-ol; p-M-1(7),8(10)-D-9-O: p-mentha-1(7),8(10)-dien-9-ol; 2,4,5-T-1,3-D: 2,4,5-trimethyl-1,3-dioxolane.

**Table 2:** Odor description and odor activity values of the most potent volatiles in turnjujube after storage at 7 °C.

Compounds	Odor Description	Aromatic Series	Odor Threshold in Water (μg·L^−1^)	OAVs of Free-Form				OAVs of Bound-Form			
				0 d	7 d	14 d	21 d	0 d	7 d	14 d	21 d
Isobutanol	Bitter, green [13]	Herbaceous [13]	6505.2 [33]	0.18	0.15	0.32	0.48	–	–	–	–
1-Butanol	Fruity, floral [34]	Fruity, floral [34]	459.2 [33]	0.32	1.22	1.52	2.16	–	–	–	–
Isopentanol	Solvent, sweet, alcohol, nail polish [35]	Chemical, sweet, fatty [35]	4 [33]	66.29	49.46	46.42	49.62	–	–	–	–
2-Methyl-1-butanol	Wine, onion [36]	Fruity	15.9 [33]	–	6.31	6.47	10.10	–	–	–	–
2,3-Butanediol	Fruity [13]	Fruity [13]	100000 [33]	–	–	0.00	0.01	–	–	–	–
1-Hexanol	Flower, green, cut grass [13]	Floral, herbaceous [13]	500 [5]	10.26	3.10	2.35	1.61	–	–	–	–
2-Phenylethanol	Floral, rose [13]	Floral [13]	564.23 [33]	0.07	0.24	0.19	0.29	0.21	0.10	0.13	0.08
***Alcohols***				***77.12***	***60.48***	***57.27***	***64.2*** ***7***	***0.21***	***0.10***	***0.13***	***0.08***
2-Butenal	Flower [36]	Floral	0.3 [33]	–	2030.90	1929.40	1477.93	–	–	–	–
Hexanal	Green [34]	Herbaceous [34]	2.4 [5]	315.54	184.02	126.41	208.39	–	–	–	–
(E)-2-Hexenal	Green [34]	Herbaceous [34]	17 [5]	17.33	12.47	5.66	–	–	–	–	–
***Aliphatic aldehydes***				***332.87***	***2227.39***	***2061.47***	***1686.32***	***0***	***0***	***0***	***0***
Acetoin	Buttery, cream [34]	Fatty [34]	14 [33]	8.04	42.58	185.13	553.29	–	–	–	–
2-Octanone	Soap, gasoline [36]	Chemical	50.2 [33]	–	0.05	0.05	0.08	–	–	–	–
***Aliphatic ketones***				***8.04***	***42.63***	***185.18***	***553.3*** ***7***	***0***	***0***	***0***	***0***
Ethyl propanoate	Fruit [36]	Fruity	10 [33]	80.12	86.71	62.41	–	–	–	–	–
Ethyl isobutyrate	Fruity [27]	Fruity [27]	0.22 [33]	30411.73	16843.59	6202.64	630.18	–	–	–	–
Isobutyl acetate	Fruit, apple, banana [36]	Fruity	25 [33]	–	1.36	1.69	3.12	–	–	–	–
Ethyl butanoate	Banana, pineapple, strawberry [35]	Fruity [35]	0.9 [33]	–	258.38	204.10	–	–	–	–	–
Ethyl isovalerate	Fruity [13]	Fruity [13]	3 [13]	272.84	191.74	92.79	37.37	–	–	–	–
Ethyl hexanoate	Fruity, apple-like [34]	Fruity [34]	5 [33]	1.32	1.43	1.56	1.63	–	–	–	–
Methyl benzoate	Sweet [11]	Sweet [11]	73 [33]	–	–	–	–	0.01	0.00	0.00	–
*n*-Hexyl butanoate	Apple peel [36]	Fruity	203 [33]	0.09	–	–	–	–	–	–	–
Methyl salicylate	Green pine [34]	Herbaceous [34]	40 [33]	–	–	–	–	0.24	0.06	–	–
Ethyl benzeneacetate	Fruit, sweet [36]	Fruity, sweet	155.55 [33]	0.55	0.50	0.34	0.40	–	–	–	–
***Esters***				***30766.65***	***17383.71***	***6565.53***	***672.7*** ***0***	***0.25***	***0.06***	***0.00***	***0***
α-Terpinene	Lemon [36]	Fruity	80 [33]	–	0.15	0.18	0.21	–	–	0.01	0.01
d-Limonene	Fruity, lemon [27]	Fruity [27]	10 [5]	–	0.78	1.16	0.90	–	–	–	–
γ-Terpinene	Fruity, lemon-like [34]	Fruity [34]	1000 [33]	0.01	0.01	0.02	0.03	–	0.00	0.00	0.00
Terpinolene	Floral, sweet, sour [5]	Floral, sweet [5]	41 [5]	–	0.14	0.16	0.19	–	–	–	–
***Monoterpene hydrocarbons***				***0.01***	***1.08***	***1.52***	***1.33***	***0***	***0.00***	***0.01***	***0.01***
(E)-Sabinene hydrate	Wood, balsamic [36]	Fatty	55000 [33]	–	0.00	0.00	0.00	–	–	–	–
Camphor	Camphor [36]	Herbaceous [27]	520 [33]	0.00	0.02	0.03	0.02	–	–	–	–
Borneol	Camphor [36]	Herbaceous [27]	140 [37]	–	–	–	0.07	29.84	16.43	11.69	19.64
4-Terpineol	Flowers, nutmeg, moldy [27]	Herbaceous, floral [27]	130 [27]	–	–	0.08	0.53	–	0.02	0.06	0.05
α-Terpineol	Oil, anise, mint [5]	Chemical [5]	330 [27]	–	–	–	–	0.01	0.01	0.01	0.01
Myrtenol	Flowery, mint [27]	Herbaceous, floral [27]	7 [33]	–	–	–	–	0.58	0.46	0.39	0.41
(E)-Carveol	Caraway, solvent [36]	Herbaceous, chemical	250 [33]	–	–	–	–	0.15	0.06	0.08	0.08
(Z)-Carveol	Caraway [35]	Herbaceous	250 [33]	–	–	–	–	0.28	0.17	0.14	0.16
***Oxygenatedmonoterpenes***				***0.00***	***0.02***	***0.11***	***0.62***	***30.86***	***17.15***	***12.37***	***20.35***
p-Ethylguaiacol	Spice, clove [36]	Floral	89.25 [33]	–	–	0.01	0.03	–	–	–	0.02
Eugenol	Clove, honey, spice [11]	Floral [11], sweet [5]	6 [5]	–	–	–	–	300.10	351.63	264.52	347.79
Isoeugenol	Clove [13]	Floral [5]	6 [13]	–	–	–	–	97.95	65.95	59.35	63.67
***Phenols***				***0***	***0***	***0.01***	***0.03***	***398.05***	***417.58***	***323.87***	***411.48***
Diethyl acetal	Fruit, cream [36]	Fruity, fatty	4.9 [33]	–	26.34	22.29	29.87	–	–	–	–
Isobutyric acid	Rancid, butter, cheese [13]	Fatty [13]	6550.5 [33]	–	0.01	0.02	–	–	–	–	–
Mesitylene	Herbaceous [38]	Herbaceous	700 [33]	0.10	0.06	0.05	0.03	–	–	–	–
***Others***				***0.10***	***26.41***	***22.36***	***29.90***	***0***	***0***	***0***	***0***

Odor description, aromatic series, and odor thresholds were obtained from reported literature. OAVs: ratio of the concentration of a molecule to its odor threshold.

## Supplementary Materials

The following are available online at https://www.mdpi.com/2304-8158/9/4/488/s1, Figure S1: The turnjujube fruit at ripening stage, Table S1: Quantitative ion, quantitative standards and calibration curves for quantification of volatile compounds in turnjujube.
