# Supplementary material for: Free and Bound Aroma Compounds of Turnjujube (Hovenia acerba Lindl.) during Low Temperature Storage
Source: Foods. 2020 Apr 13;9(4):488. doi: 10.3390/foods9040488 (PMC7230446; doi:10.3390/foods9040488)
Supplement: Supplementary file 1 [file foods-09-00488-s001.pdf]

Supplementary Materials:

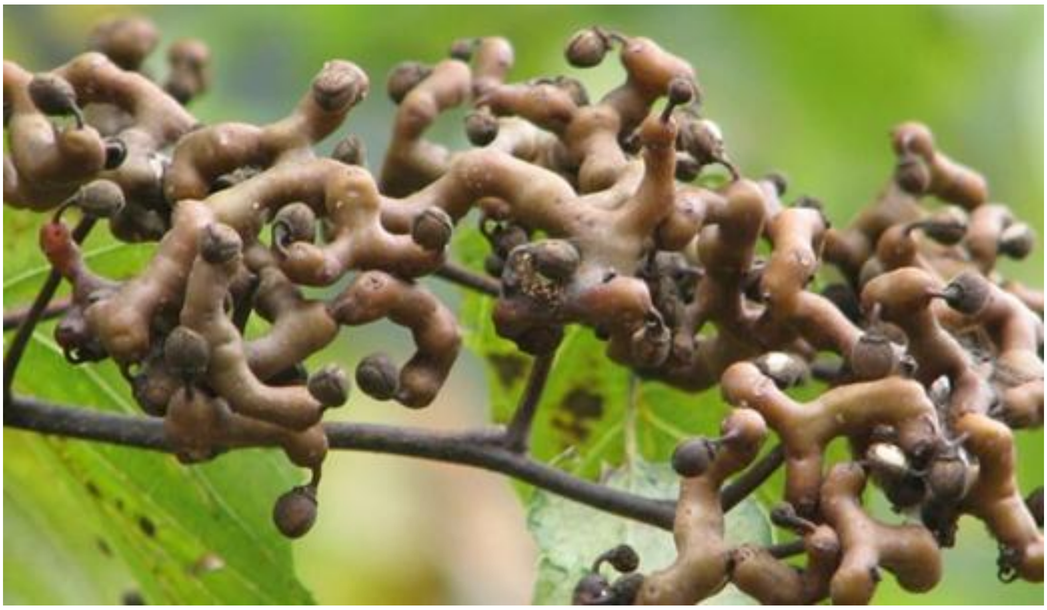

Figure S1. The turnjujube fruit at ripening stage.

Table S1. Quantitative ion, quantitative standards, and calibration curves for quantification of volatile compounds in turnjujube.

Compounds	CAS Nos.	Quantitative Ion (*m/z*)	Quantitative standards	Calibration curves	R^2	Linear range (μg L^{-1})
Isobutanol	78-83-1	43	Isobutanol	y=2495.55x+129.28	0.9756	40.71- 3700.90
2-Butenal	4170-30-3	70	(E)-2-Hexenal	y=2507.65x-31.58	0.9983	8.17- 742.47
(E)-2-Hexenal	6728-26-3	55	(E)-2-Hexenal	y=2507.65x-31.58	0.9983	8.17- 742.47
1-Butanol	71-36-3	56	1-Butanol	y=2902.83x-80.06	0.9820	11.71- 1064.93
3-Hydroxybutanal	107-89-1	43	Acetoin	y=2512.81x+28.42	0.9960	98.44- 8948.87
Acetoin	513-86-0	88	Acetoin	y=2512.81x+28.42	0.9960	98.44- 8948.87
Ethyl propanoate	105-37-3	57	Ethyl propanoate	y=1931.13x+82.75	0.9770	12.44- 1130.48
2,4,5-Trimethyl-1,3-dioxolane	3299-32-9	43	Diethyl acetal	y=2208.55x+6.58	0.9927	2.02- 183.25
Diethyl acetal	105-57-7	45	Diethyl acetal	y=2208.55x+6.58	0.9927	2.02- 183.25
Isopentanol	123-51-3	55	Isopentanol	y=3673.36x-155.83	0.9970	3.57- 324.14
2-Methyl-1-butanol	137-32-6	57	2-Methyl-1-butanol	y=1472.93x+6.69	0.9852	3.5- 318.42
Isobutyric acid	79-31-2	73	Isobutyric acid	y=2876.63x-9.15	0.9891	1.73- 157.44
Ethyl isobutyrate	97-62-1	43	Ethyl isobutyrate	y=1835.47x-1091.46	0.9906	136.92- 12447.24
Isobutyl acetate	110-19-0	43	Isobutyl acetate	y=1074.67x-34.00	0.9915	3.51- 319.06
2,3-Butanediol	513-85-9	45	2,3-Butanediol	y=2441.00x-58.29	0.9754	10.36- 942.18
Hexanal	66-25-1	56	Hexanal	y=2041.16x-40.33	0.9836	13.55- 1231.47
Ethyl butanoate	105-54-4	71	Ethyl butanoate	y=1811.65x-34.08	0.9773	4.73- 430.00
Ethyl 2-butenoate	623-70-1	69	Ethyl 2-butenoate	y=998.67x-392.97	0.9772	51.84- 4712.94
Ethyl isovalerate	108-64-5	88	Ethyl isovalerate	y=1215.58x-15.22	0.9799	21.83- 1984.63
Ethyl 3-hydroxy-3-methylbutanoate	18267-36-2	43	Ethyl isovalerate	y=1215.58x-15.22	0.9799	21.83- 1984.63

1-Hexanol	111-27-3	56	1-Hexanol	y=2003.40x+257.13	0.9832	76.01- 6910.38
Butyl isobutyrate	97-87-0	71	Butyl isobutyrate	y=725.97x+2.57	0.9906	0.72- 65.37
Mesitylene	108-67-8	105	Mesitylene	y=1085.92x-5.32	0.9965	2.22- 201.54
2-Octanone	111-13-7	58	2-Octanone	y=64.20x+0.27	0.9826	0.16- 4.81
Ethyl hexanoate	123-66-0	88	Ethyl hexanoate	y=445.90x+1.52	0.9961	0.48- 43.79
α-Terpinene	99-86-5	121	α-Terpinene	y=232.03x+7.09	0.9897	1.38- 125.65
D-Limonene	5989-27-5	93	D-Limonene	y=282.40x+5.26	0.9817	0.79- 72.18
γ-Terpinene	99-85-4	121	γ-Terpinene	y=205.86x+8.77	0.9931	2.09- 298.89
Terpinolene	586-62-9	121	Terpinolene	y=215.06x+4.07	0.9790	0.55- 50.04
Methyl benzoate	93-58-3	105	Methyl benzoate	y=7.94x+2.04	0.9900	1.73- 19.06
6-Camphenol	3570-04-5	108	(E)-Pinocarveol	y=2246.90x+0.29	0.9828	1.70- 154.83
(E)-Pinocarveol	547-61-5	92	(E)-Pinocarveol	y=2246.90x+0.29	0.9828	1.70- 154.83
(E) - Sabinene hydrate	17699-16-0	93	(E)-Pinocarveol	y=2246.90x+0.29	0.9828	1.70- 154.83
Ethyl 2,4-hexadienoate	2396-84-1	95	Ethyl 2,4-hexadienoate	y=532.22x-9.77	0.9773	2.15- 195.87
2-Phenylethanol	60-12-8	91	2-Phenylethanol	y=1173.36x-11.11	0.9856	4.74- 1431.2
Camphor	464-48-2	81	Camphor	y=631.95x-2.77	0.9816	0.94- 85.86
(E)-Carveol	1197-07-5	119	(E)-Carveol	y=2161.01x+18.46	0.9916	2.94-907.44
(Z)-2-(3,3-Dimethylcyclohexylidene)ethanol	26532-23-0	69	(E)-Carveol	y=2161.01x+18.46	0.9916	2.94-907.44
(Z)-Carveol	1197-06-4	119	(E)-Carveol	y=2161.01x+18.46	0.9916	2.94-907.44
Isogeraniol	5944-20-7	109	(E)-Carveol	y=2161.01x+18.46	0.9916	2.94-907.44
5-Caranol	6909-21-3	93	(E)-Carveol	y=2161.01x+18.46	0.9916	2.94-907.44
2,7-Dimethylocta-2,6-dienol	22410-74-8	69	(E)-Carveol	y=2161.01x+18.46	0.9916	2.94-907.44

(Z)-p-Mentha-2,8-dien-1-ol	3886-78-0	119	(E)-Carveol	y=2161.01x+18.46	0.9916	2.94-907.44
p-Mentha-1(7),8(10)-dien-9-ol	29548-13-8	79	(E)-Carveol	y=2161.01x+18.46	0.9916	2.94-907.44
(Z)-Verbenol	18881-04-4	91	cis-Verbenol	y=2817.24x-33.75	0.9829	2.94- 267.33
n-Hexyl butanoate	2639-63-6	43	n-Hexyl butanoate	y=664.83x+1.25	0.9845	0.87- 79.04
Borneol	10385-78-1	95	4-Terpineol	y=258.95x-31.31	0.9947	9.27- 1274.92
Borneol	10385-78-1	95	4-Terpineol	y=1204.35x-8865.05	0.9880	1274.92- 115901.52
4-Terpineol	562-74-3	93	4-Terpineol	y=258.95x-31.31	0.9947	9.27- 1274.92
α-Terpineol	98-55-5	93	α-Terpineol	y=2535.63x-32.52	0.9903	1.05- 95.76
Methyl salicylate	119-36-8	120	Methyl salicylate	y=355.90x+16.60	0.9852	7.86- 714.41
Myrtenol	515-00-4	79	Myrtenol	y=362.73x-8.70	0.9837	4.71- 427.73
Verbenone	1196-01-6	107	Verbenone	y=2193.47x+5.25	0.9926	2.42- 219.55
2,6,8-trimethylnonan-4-one	123-18-2	57	Verbenone	y=2193.47x+5.25	0.9926	2.42- 219.55
Linalyl formate	115-99-1	69	Ethyl benzeneacetate	y=1100.69x+13.47	0.9820	2.04- 185.23
Ethyl benzeneacetate	101-97-3	91	Ethyl benzeneacetate	y=1100.69x+13.47	0.9820	2.04- 185.23
p-Ethylguaiacol	2785-89-9	137	p-Ethylguaiacol	y=74.75x+0.50	0.9963	0.53- 106.62
Eugenol	97-53-0	164	Eugenol	y=1028.74x-1.98	0.9847	7.25- 2659.19
Isoeugenol	97-54-1	164	Isoeugenol	y=2108.63x+60.12	0.9799	8.07- 733.54

Quantitative ion used for peak area evaluation of compounds.
y: concentration in μg L^{-1}.
x: peak area ratio of a compound to the internal standard (4-methyl-2-pentanol).
R^2: Regression coefficient.
